# A Phase 1 Study to Assess the Pharmacokinetics, Food Effect, Safety, and Tolerability of Sepiapterin in Healthy Japanese and Non-Japanese Participants

**DOI:** 10.3390/ph17111411

**Published:** 2024-10-22

**Authors:** Lan Gao, Diksha Kaushik, Kimberly Ingalls, Neil Smith, Ronald Kong

**Affiliations:** PTC Therapeutics, Warren, NJ 07059, USA; dkaushik@ptcbio.com (D.K.); kingalls@ptcbio.com (K.I.); nsmith@ptcbio.com (N.S.); rkong@ptcbio.com (R.K.)

**Keywords:** sepiapterin, tetrahydrobiopterin, BH_4_, phenylketonuria (PKU), pharmacokinetics (PK), food effects, Japanese

## Abstract

Background: Sepiapterin is a natural precursor of tetrahydrobiopterin (BH_4_), a key cofactor for phenylalanine hydroxylase. It is being developed for the treatment of patients with phenylketonuria. In this study, the ethnic differences in pharmacokinetics and safety of sepiapterin in Japanese and non-Japanese participants and food effects were evaluated. Methods: Healthy participants (*n* = 60) received a single oral dose of sepiapterin at either 20, 40, or 60 mg/kg with a low-fat diet. The Japanese participants received two doses at 40 mg/kg, either under fasted conditions or with a low-fat diet with a 3-day washout period in between. Results: Sepiapterin was well tolerated in all participants, with no serious adverse events. Sepiapterin was quickly absorbed (T_max_ 1.4–4.5 h) and rapidly and extensively converted to BH_4_ (T_max_ ~4 h). Exposures to sepiapterin were <1% of BH_4_. BH_4_ exposures were essentially dose-independent between 20 and 60 mg/kg. A low-fat diet increased BH_4_ exposures in Japanese participants by 1.7-fold compared with fasted conditions. Conclusions: BH_4_ exposures (C_max_ and AUC_0–last_) in Japanese participants were 10–30% higher than in non-Japanese participants, which is deemed not clinically relevant; no dose adjustment is warranted. The slightly higher BH_4_ exposures in Japanese participants are likely due to the higher frequency of *ABCG2* c.421C>A mutation in the Japanese population.

## 1. Introduction

Phenylketonuria (PKU) is a rare inborn error of metabolism disease caused by reduced functionality of phenylalanine hydroxylase (PAH), an essential enzyme converting phenylalanine (Phe) to tyrosine [1,2]. PAH with its cofactor tetrahydrobiopterin (BH_4_) metabolizes Phe to tyrosine. In PKU, where PAH is deficient, elevated blood Phe levels and corresponding increased Phe in the brain can result in neurocognitive deficits [3]. PKU is diagnosed at birth with the near-universal adoption of newborn screening. Currently, there is no cure for PKU. The initial management of PKU consists of a prompt institution of a stringent dietary Phe restriction supplemented with specifically designed low-Phe medical formula (amino acid powder) and foods. However, dietary management alone faces great challenges in adherence, especially in older children, adolescents, and adults where the Phe-restricted diet causes nutritional inadequacy and social isolation.

Sepiapterin is a novel oral small molecule currently in clinical development for the treatment of pediatric and adult patients with PKU. Sepiapterin is a natural precursor of BH_4_, and its administration enhances BH_4_ concentrations. Sepiapterin and BH_4_ both bind to PAH to correct and stabilize the conformational structure of PAH, hence enhancing the activity of variant PAH. The binding of either BH_4_ or sepiapterin and the effects on the PAH enzyme are PAH variant-specific [4,5]. Sepiapterin has been shown to efficiently reduce blood Phe in Phase 2 and Phase 3 studies in patients with PKU [6,7]. In the recently completed Phase 3 APHENITY study (NCT05099640), children and adult participants with PKU were treated with orally administered sepiapterin at 60 mg/kg/day in a fed state and showed clinically significant benefits [7]. Of 156 participants, 114 (73%) responded to sepiapterin treatment (the response was defined as a reduction of ≥15% blood Phe level from baseline). A significant reduction of 64.2% from the baseline blood Phe concentration was observed among 98 participants in the primary analysis set, which included all participants 2 years and older who achieved ≥30% reduction in blood Phe following the 14-day sepiapterin responsiveness test during Part 1 of the study.

The pharmacokinetics (PK) of sepiapterin and its metabolite BH_4_ were previously examined in adult healthy volunteers in a first-in-human study following single and multiple ascending oral doses of sepiapterin and in a relative bioavailability and food effects study [2,8]. Oral sepiapterin is quickly absorbed and rapidly and extensively converted to BH_4_. The sepiapterin maximum-observed plasma concentration (C_max_) and area under the plasma concentration–time curve from the start of dosing to the last quantifiable concentration (AUC_0–last_) were generally <1% of the BH_4_ values in healthy volunteers. Plasma BH_4_ reached C_max_ around 4 h (T_max_) and returned to near-baseline endogenous concentrations by 24 h. Administering sepiapterin with low-fat and high-fat diets increased BH_4_ AUC from the start of dosing to 24 h (AUC_0–24h_) by 1.7-fold and 2.8-fold, respectively. No apparent accumulation of BH_4_ was observed after 7-day repeated daily dosing at doses up to 60 mg/kg with a high-fat diet. BH_4_ exposures increased approximately dose proportionally within the dose range of 5 to 20 mg/kg and far less than dose proportionally in the dose range of 20 to 60 mg/kg when administered with a high-fat diet. The highest doses that have been studied in healthy volunteers and participants with PKU are a single dose of 80 mg/kg under fasted conditions and repeated doses of 60 mg/kg QD (once daily) with a high-fat diet [2,8].

PKU has been described in all ethnic groups, and its incidence worldwide varies widely. It was estimated that 0.45 million individuals have PKU worldwide [9]. In Japan, it occurs in approximately 1 in every 125,000 subjects [9]. To shorten the delay of introduction of innovative drugs into Japan, it is recommended in ICH E5(R1) and Japan PMDA (PSB/PED Notification No. 1225-2) to investigate potential interethnic differences by enrolling Japanese participants into multi-regional clinical trials (MRCTs).

In all previous clinical studies of sepiapterin, both in healthy volunteers and patients with PKU, the majority of participants were white; only a very limited number were Asian. The aim of this Phase 1 study was to evaluate potential differences in the PK and safety profile of sepiapterin in Japanese participants compared to non-Japanese participants. The PK profile, food effect, and safety and tolerability of three doses of sepiapterin (20, 40, and 60 mg/kg) in Japanese and non-Japanese healthy participants were evaluated. These doses were selected as these were the doses being investigated in the Phase 3 studies in patients with PKU 2 years and older.

Administering sepiapterin with food increases exposure to sepiapterin and BH_4_ [8]. Hence, PKU patients have generally been instructed to take sepiapterin with food during treatment. The fat composition of the Phe-restricted diet for PKU patients is similar to that of a low-fat diet. Hence, during this study, sepiapterin was administered with a low-fat meal in all cohorts. In the cohort with the 40 mg/kg dose, all Japanese participants, received an additional dose under the fasted condition to further explore the food effect in the Japanese population.

## 2. Results

### 2.1. Demographic Characteristics

A total of 60 participants enrolled and completed the study. Errors in blood sample collection were identified after completion of Cohorts 1 and 2, and therefore, additional participants were enrolled in Cohorts 4 and 5 as replacements. All 60 participants were included in the safety analysis set, and only participants (*n* = 36) in Cohorts 3, 4, and 5 were included in the PK analysis set. Of the thirty-six participants (eighteen Japanese and eighteen non-Japanese) enrolled in Cohorts 3, 4, and 5 for PK assessment, two participants (Japanese) were excluded from the PK analysis for the fed condition due to deviation from the low-fat diet on the day of dosing.

The mean age of participants was 32.0 years (range 20–55 years), the mean body weight was 66.4 kg (range 47.6–90.1 kg), and the mean BMI was 22.9 kg/m^2^ (range 18.5–30.0 kg/m^2^) (Table 1). Comparable numbers of male (*n* = 34) and female (*n* = 26) participants were enrolled. Baseline demographics were generally similar between Japanese and non-Japanese participants.

### 2.2. Pharmacokinetics

Predose endogenous sepiapterin concentrations in plasma were below the lower limit of quantitation (LLOQ, 0.75 ng/mL), while the mean endogenous BH_4_ concentrations in plasma were in the range of 2.01 to 2.95 ng/mL (Table 2). Small fluctuations of endogenous plasma BH_4_ concentration were observed, with slightly higher concentrations in the afternoon compared to in the morning (Table 2). The endogenous BH_4_ in Japanese participants (2.39 and 2.95 ng/mL in the morning and afternoon, respectively) was slightly higher than in non-Japanese participants (2.01 and 2.50 ng/mL in the morning and afternoon, respectively). Given that the baseline level of endogenous BH_4_ was considered relatively negligible compared to BH_4_ C_max_ following sepiapterin administration (<5%), the PK parameters of BH_4_ without baseline correction are presented here.

**Figure 1:**
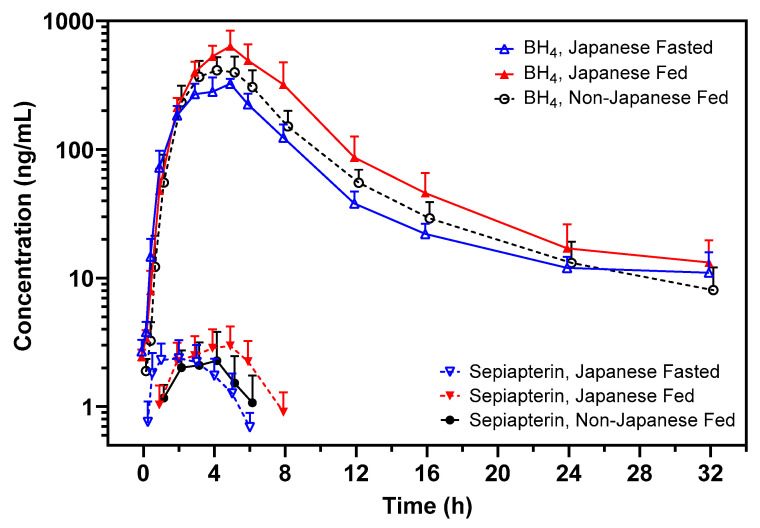
Mean (SD) sepiapterin and BH_4_ concentrations following oral administration of 40 mg/kg Sepiapterin in non-Japanese and Japanese participants. BH_4_, tetrahydrobiopterin; SD, standard deviation. Non-Japanese participants received the dose with a standard low-fat low-calorie meal. Japanese participants received two single doses separated by a 4-day washout in between either under a fasted condition or with a low-fat low-calorie meal in each period in their specific treatment sequence.

Sepiapterin (20 to 60 mg/kg dose range) was quickly absorbed following oral administration with C_max_ (2.1 to 4.9 ng/mL) reached between 1.0 and 5.0 h (T_max_) (Table 3). Absorbed sepiapterin was rapidly and extensively converted to BH_4_, and the sepiapterin concentrations in plasma dropped below the LLOQ by 12 h for the majority of participants (Figure 1). Sepiapterin exposures (C_max_ and AUC_0–last_) were generally <1% of its metabolite BH_4_ (Table 3). Due to the rapid elimination, sepiapterin T_1/2_ and AUC_0–inf_ could not be reliably estimated.

BH_4_ C_max_ in plasma was reached between 4 and 5 h (T_max_) (Table 3), and concentrations declined steadily following the T_max_. In some individuals, this decline was biphasic, with the second phase starting approximately between 12 and 24 h post-dose (Figure 1). As a result, the terminal T_1/2_ for BH_4_ could not be reliably estimated for some participants due to the insufficient number of time points available in the terminal phase. For participants with estimable terminal T_1/2_, mean values ranged between 6 and 7.6 h (Table 3).

#### 2.2.1. Ethnic Comparison

There was no apparent difference in the absorption rate between Japanese and non-Japanese participants, and the T_max_ for BH_4_ ranged between 3.0 and 5.0 h for both populations (Table 3).

BH_4_ exposure was slightly higher in Japanese participants compared with non-Japanese participants; GMRs (%) of BH_4_ exposures (C_max_, AUC_0–last_, and AUC_0–inf_) were between 110% and 129% (Table 4). This difference is not considered clinically relevant, and therefore, no dose adjustment is required for Japanese participants.

#### 2.2.2. Food Effects

**Figure 2:**
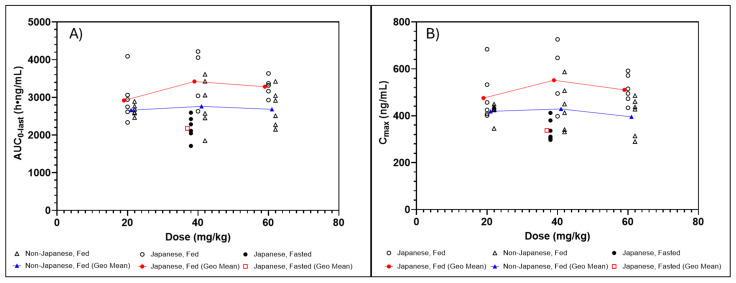
Dose linearity and food effects of BH_4_ AUC_0–last_ (**A**) and C_max_ (**B**) following sepiapterin administration in non-Japanese and Japanese participants. AUC_0–last_, area under the plasma concentration–time curve from the start of dosing to the last quantifiable concentration; BH_4_, tetrahydrobiopterin; C_max_, maximum observed plasma concentration.

In Japanese participants, the administration of sepiapterin with food increased BH_4_ exposures relative to fasted conditions (Table 5). BH_4_ AUC_0–last_ and C_max_ were approximately 1.7-fold higher with a low-fat diet compared with fasted conditions (Figure 2).

#### 2.2.3. Dose Proportionality

No differences in dose proportionality were observed between Japanese and non-Japanese participants. In the dose range of 20 to 60 mg/kg of sepiapterin, negligible increases in BH_4_ exposure were observed with an increased dose. Using the power model, the slope (90% CI) for natural log-transformed BH_4_ AUC_0–inf_, AUC_0–last_, and C_max_ was 0.026 (−0.094, 0.145), 0.064 (−0.042, 0.170), and 0.017 (−0.101, 0.134), respectively (Figure 2). The 90% CI for all three parameters contained zero. Similarly, negligible increases in sepiapterin exposure were observed with increased sepiapterin dose.

### 2.3. Safety

No deaths or serious AEs occurred during this study, and no participant withdrew due to a TEAE. The incidence of TEAEs was comparable between Japanese and non-Japanese participants, and there was no relationship with sepiapterin dose (Table 6). A total of 18 participants (30%) experienced 33 TEAEs. Of the thirty-three TEAEs, twenty-eight were mild (Grade 1) and five were moderate (Grade 2) in severity. Headache was the most commonly reported TEAE (four (13.3%) Japanese participants and three (10.0%) non-Japanese participants). Of the TEAEs, 14 were considered probably or possibly related to sepiapterin. There were no clinically relevant changes in laboratory evaluations, vital signs, and 12-lead ECG measurements.

## 3. Discussion

Exposure to BH_4_ was slightly higher in Japanese participants than in non-Japanese participants, and sepiapterin and BH_4_ PK parameters were generally comparable with previous studies in which the majority of participants were white [2,8]. Sepiapterin was rapidly absorbed, quickly and extensively converted to BH_4_, and declined to below the LLOQ generally by 12 h postdose. The C_max_ and AUC_0–last_ of sepiapterin were generally <1% of those of BH_4_, with BH_4_ having a terminal T_1/2_ of approximately 6 h in most participants.

In this study, BH_4_ exposure was found to be independent of sepiapterin dose (20 to 60 mg/kg) in both Japanese and non-Japanese participants. This deviated from previous clinical studies conducted in the same sepiapterin dose range, in which an increase in BH_4_, albeit far less than dose-proportional, was observed [2,8]. However, small, less than dose-proportional increases in BH_4_ exposure were observed in the dose range of 20 to 40 mg/kg sepiapterin in Japanese participants in this study. For Japanese participants, BH_4_ C_max_ and AUC_0–last_ increased from 485 to 567 ng/mL and 2830 to 3490 h·ng/mL, respectively, representing an increase of 16.9% and 17.5% when the dose was increased from sepiapterin 20 to 40 mg/kg administered with a low-fat diet. This degree of increase in BH_4_ is consistent with clinical studies in which most participants were white [2,8]. In a previous study, in which 78% of participants were white, BH_4_ C_max_ and AUC_0–24h_ increased from 317 to 519 ng/mL and 2010 to 3140 h·ng/mL, respectively, when the dose, administered with a low-fat low-calorie meal, was increased from 20 to 60 mg/kg [8]. This represents an increase of 63.7% in BH_4_ C_max_ and 56.2% in BH_4_ AUC_0–24h_ with a 3-fold increase in sepiapterin dose. The independence of BH_4_ exposures from doses observed in this study, across the sepiapterin dose range from 20 to 60 mg/kg, suggests that the absorption of the drug was likely saturated at the higher end of the dose range and there was only a negligible difference in exposure without an overall central trend, considering the interindividual variability and the small sample size.

PK analysis demonstrated that BH_4_ exposures were slightly higher (10% to 29%) in Japanese participants compared with non-Japanese participants. However, this modest increase is deemed not clinically relevant, and a dose adjustment is not required for the Japanese population. The higher BH_4_ exposures in Japanese participants could potentially be attributed to a higher frequency of the *ABCG2* c.421C>A polymorphism in Asian populations.

Both sepiapterin and BH_4_ have been shown to be substrates of the breast cancer resistance protein (BCRP), also known as the ABCG2 transporter. It is known that polymorphisms in BCRP contribute to interindividual differences in exposures to orally administered drugs that are substrates of BCRP [10,11,12,13,14]. It is hypothesized that the slightly higher BH_4_ exposures in Japanese participants observed in this study could be due to the higher reported frequency of the *ABCG2* c.421C>A mutation present in Eastern Asian populations (34% to 45%) in comparison to white populations (11% to 17%) [15,16,17], although the BCRP genotype was not collected in this study to confirm this hypothesis. Clinical studies have demonstrated that people have increased exposures to rosuvastatin, an indicator substrate of BCRP, when having the BCRP genotype *ABCG2* c.421C/C, c.421C/A, and c.421A/A, in that order. Thus, the higher exposures of rosuvastatin and some other BCRP substrates, such as sulfasalazine, fluvastatin, atorvastatin, and sunitinib [18], in Eastern Asians, such as Japanese and Chinese subjects, are attributed to allele frequency in BCRP mutations.

In a drug–drug interaction study of sepiapterin, it was found that BH_4_ C_max_, AUC_0–last_, and AUC_0–inf_ in participants with the *ABCG2* c.421C/A genotype were 1.36-fold, 1.39-fold, and 1.38-fold higher, respectively, than in participants with the c.421C/C genotype [19]. The C_max_, AUC_0–last_, and AUC_0–inf_ for rosuvastatin were found to be 1.60-fold, 1.61-fold, and 1.52-fold higher, respectively, in participants with the c.421C/A genotype compared to participants with the c.421C/C genotype in the same study [19].

It is proposed that the BCRP genotype information be collected in future clinical studies of sepiapterin to explore the reasons for the difference in BH_4_ exposures between Japanese and non-Japanese populations, or more widely, between Eastern Asian and white populations.

If such inter-ethnic differences in BH_4_ exposures are confirmed to be due to the *ABCG2* c.421C>A polymorphism, sepiapterin has the potential to be a better clinical indicator compound for pharmacogenomic/pharmacogenetic studies than the currently widely used rosuvastatin for the following reasons: sepiapterin is not a substrate of OATP1B1 (organic anion transporting peptide member 1B1), so it is unlikely to be impacted by OATP1B1 polymorphism as is the case with rosuvastatin; there is negligible biliary excretion of sepiapterin, as demonstrated in a ^14^C-labeled rat mass balance study (data on file); and sepiapterin metabolism is mainly mediated by sepiapterin reductase, dihydrofolate reductase, and aromatic amino acid hydroxylases, and therefore, it is unlikely to be impacted by polymorphisms of CYP2C9 or CYP3A4/5 [1].

Examination of the PK of BH_4_ in previous clinical studies has shown a monophasic decline of BH_4_ concentrations up to 24 h post-dose [2]. However, in this study reported here, where PK samples were collected up to 32 h post-dose, a biphasic decline in BH_4_ concentrations post-T_max_ was observed in some participants, with the second phase starting approximately 12 to 24 h post-dose. Due to insufficient duration of blood sample collection, the T_1/2_ could not be reliably estimated for these participants; however, if the T_1/2_ of the second phase were to be calculated, the value could be as high as 49.6 h (with a span of 0.322). It is postulated that the second phase of elimination is likely related to the release of BH_4_ trapped deep in peripheral tissues. This appears to have a minimal impact on the overall BH_4_ exposures, given no apparent accumulation of BH_4_ has been consistently demonstrated in healthy adults and patients with PKU at repeated doses of sepiapterin up to 60 mg/kg administered with food [2,8].

Examination of the PK of BH_4_ in Japanese participants following administration of sepiapterin 40 mg/kg with a low-fat low-calorie meal showed that exposures (C_max_ and AUC_0–t_) were increased by approximately 1.7-fold compared with fasted conditions. This is consistent with observations in other ethnic populations (mainly participants who were white) where administration of sepiapterin with a low-fat diet increased BH_4_ exposures by approximately 1.7-fold and with a high-fat diet increased BH_4_ exposures by approximately 2.8-fold [8].

## 4. Methods

### 4.1. Ethics

This Phase 1 study was conducted at a single clinical research facility managed by LabCorp (Leeds, UK) in full compliance with the protocol, and the principles of the International Council for Harmonization (ICH) Good Clinical Practice (GCP), the Declaration of Helsinki, and applicable Medicines and Healthcare Products Regulatory Agency (MHRA) regulatory requirements. The study protocol was approved by the Independent Ethics Committees (IEC) of Fast Track REC (Research Ethic Committee), London, UK; London-Harrow REC, Bristol, UK; and the MHRA prior to initiation. All participants provided written informed consent prior to entering the study.

### 4.2. Study Design

**Figure 3:**
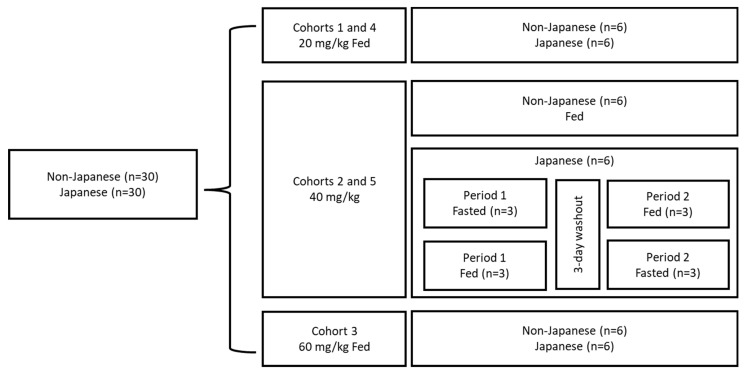
Study design. Fed: low-fat low-calorie diet Cohorts 4 and 5 were replacements for Cohorts 1 and 2 due to errors during blood sample collection for Cohorts 1 and 2 that resulted in non-evaluable PK data.

This was a Phase 1, open-label, single-dose study of sepiapterin in 60 healthy adult males and females (30 Japanese and 30 non-Japanese) equally divided into five cohorts, with each cohort composed of six Japanese and six non-Japanese participants (Figure 3). Participants received a single dose of sepiapterin at 20 mg/kg in Cohorts 1 and 4, 40 mg/kg in Cohorts 2 and 5, and 60 mg/kg in Cohort 3. Sepiapterin was dosed with a low-fat diet with compositions consistent with the US Food and Drug Administration (FDA) guidance for all participants in Cohorts 1, 3, and 4, and all non-Japanese participants in Cohorts 2 and 5 [20]. Japanese participants in Cohorts 2 and 5 were randomized into one of the two sequences, and sepiapterin (40 mg/kg) was administered either with a low-fat diet or under the fasted condition in one of the two periods with a 3-day washout period in between. Cohorts 4 and 5 were the replacements for Cohorts 1 and 2 due to errors during blood sample collection for Cohorts 1 and 2 that resulted in non-evaluable PK data.

All participants fasted overnight for at least 10 h and received sepiapterin in either a fasted condition or fed state (within 30 min after starting the breakfast (low-fat low-calorie diet) and 5 min after consumption of food). Intake of food was restricted for at least 4 h post-dose, and fluid intake was restricted from 1 h prior to dose to 2 h post-dose (except for those foods and liquids provided in the meal and provided for dose administration). Additional restrictions included no consumption of xanthine/caffeine from 24 h prior to dose to 24 h post-dose and alcohol from 48 h prior to dosing to discharge from the clinical site.

### 4.3. Participants

For this study, 30 healthy Japanese participants and 30 healthy non-Japanese participants were enrolled across the cohorts (Figure 3). Key inclusion criteria included the following: healthy adult male and female participants aged 18–55 years (inclusive) with a body mass index (BMI) ranging from 18.5 to 30.0 kg/m^2^ (inclusive). Japanese participants must have had two Japanese biological parents and four Japanese grandparents, not lived outside of Japan for more than 10 years at the time of screening, and had no significant change in diet since leaving Japan. For non-Japanese participants, it was required that they were of European, white Latin American, or African descent. Key exclusion criteria included pregnancy, lactation, or suffering from any medical condition that could confound the results of the study. Females were required to have a negative pregnancy test at screening and be either abstinent from sex or use at least 2 forms of contraception during the study.

Matching of Japanese and non-Japanese participants occurred at the cohort level on sex, cohort mean age (within ±10 years), and BMI (within ±6 kg/m^2^) at the screening visit.

### 4.4. Pharmacokinetic Sample Collection and Bioanalytical Methods

Whole blood samples (3.0 mL each) were obtained on the day prior to each sepiapterin administration (between 2:00 p.m. and 8:00 p.m. (BH4 only)), pre-dose (within 1 h before dosing), and 0.25, 0.5, 1, 2, 3, 4, 5, 6, 8, 12, 16, 24, and 32 h post-dose via venipuncture into Vacutainer^®^ tubes prefilled with K_2_EDTA as anticoagulant and put on wet ice. Detailed procedures regarding the collection, storage of plasma samples, and quantitative determination of sepiapterin and BH_4_ in plasma using a validated HPLC-MS/MS method were described previously [21].

### 4.5. Data Analysis

#### 4.5.1. Safety and Tolerability

All adverse events (AEs) were coded using the Medical Dictionary for Regulatory Activities version 24.1. All AEs were assigned a severity grade using Common Terminology Criteria for Adverse Events (CTCAE) Version 5.0. Treatment-emergent adverse events (TEAEs) and drug-related AEs were assessed daily for the duration of the study and at the safety follow-up visit. Evaluations included clinical laboratory tests (chemistry, hematology, urinalysis), physical examination, 12-lead electrocardiograms (ECGs), and vital signs (blood pressure, respiratory rate, body temperature, and heart rate).

#### 4.5.2. Statistical Analyses

No formal sample size calculations were performed; the number of participants (six Japanese and six non-Japanese/cohort) was chosen based on feasibility.

Non-compartmental analysis of plasma concentration–time data was performed using Phoenix^®^ WinNonlin, version 8.1.1 or higher (Certara, Princeton, NJ, USA). AUC was calculated using the linear up logarithm–down trapezoidal method. Regression was performed on the terminal elimination phase of the individual semi-logarithm concentration-time data. At least three non-zero post-T_max_ concentrations, the span ≥ 1.5-fold of the estimated T_1/2_, and adjusted R^2^ ≥ 0.80 were required for the regression. AUC_0–inf_ was estimated only if T_1/2_ could be reliably determined, and the extrapolated AUC was ≤20%.

For analysis of ethnic variance between Japanese and non-Japanese participants, the C_max_, AUC_0–last_, and AUC_0–inf_ of sepiapterin and BH_4_ were analyzed using an analysis of variance (ANOVA) model following natural log-transformation of PK parameters. The model included treatment group and race as fixed effects, whereas the participant was a random effect. For each PK parameter, the LSM (least square mean) for each group, the difference in LSMs between Japanese and non-Japanese participants, and corresponding 90% confidence intervals (CIs) were calculated. These values were back-transformed to calculate the geometric least square mean (GLSM), geometric mean ratio (GMR) of GLSMs, and corresponding 90% CI for the ratio of GLSM.

The effect of food on PK parameters (C_max_, AUC_0–last_, and AUC_0–inf_) in Japanese participants was assessed using the same mixed-effects model as described for the ethnic analysis, with feeding condition (fasted or fed) as a fixed effect instead of race.

Dose proportionality was explored using the power model that included race as a fixed effect with natural logarithm-transformed PK parameters (C_max_, AUC_0–last_, and AUC_0–inf_) [22].

ln(parameter) = intercept + slope × ln(dose) + race + error

Dose proportionality was concluded if the 90% CI for the slope of regression was within the range of 0.80 and 1.25, and dose independence was concluded if the 90% CI contained zero.

## 5. Conclusions

Sepiapterin was well tolerated by both Japanese and non-Japanese healthy adults following single oral doses of up to 60 mg/kg. All TEAEs were mild (twenty-eight out of thirty-three) or moderate (five out of thirty-three) in severity. BH_4_ exposures in Japanese participants were 10% to 29% higher than in non-Japanese participants following oral administration of sepiapterin in the dose range of 20 to 60 mg/kg with a low-fat diet; however, this is not deemed to be clinically relevant, and no dose adjustment is required for Japanese participants. The slightly higher BH_4_ exposures in Japanese participants are hypothesized to be due to the higher frequency of the *ABCG2* c.421C>A mutation in the Japanese population. In Japanese participants, BH_4_ exposures were 1.7-fold higher when sepiapterin was administered at 40 mg/kg with a low-fat diet compared with fasted conditions. The increase in BH_4_ exposures was negligible in the sepiapterin dose range of 20 to 60 mg/kg.

## Figures and Tables

**Table 1:** Baseline demographics of study population.

Variable	PK Population (*n* = 36) ^a^		Overall ^b^ (*n* = 60)
	Non-Japanese (*n* = 18)	Japanese (*n* = 18)	
Age (years), Median (min, max)	31.0 (20, 54)	35.5 (25, 51)	32.0 (20, 55)
Sex, *n* (%)			
Male	11 (61.1)	10 (55.6)	34 (56.7)
Female	7 (38.9)	8 (44.4)	26 (43.3)
Race, *n* (%)			
Asian	0	18 (100)	30 (50.0)
White	15 (83.3)	0	26 (43.3)
Black or African American	2 (11.1)	0	3 (5.0)
Other	1 (5.6)	0	1 (1.7)
Body weight (kg), mean (min, max)	74.0 (56.1, 90.1)	61.7 (47.6, 81.2)	66.4 (47.6, 90.1)
BMI (kg/m^2^), mean (min, max)	24.4 (20.4, 28.2)	21.5 (18.8, 25.6)	22.9 (18.5, 30.0)

BMI, body mass index; PK, pharmacokinetics. ^a^ Participants with valid concentration measurements for PK evaluation from Cohorts 3, 4, and 5. ^b^ All participants enrolled in the study from Cohorts 1, 2, 3, 4, and 5.

**Table 2:** Endogenous BH_4_ plasma concentrations in Japanese and non-Japanese participants.

Parameter	Japanese		Non-Japanese	
	p.m.	a.m.	p.m.	a.m.
*n*	17	18	18	18
Mean (SD)	2.95 (0.667)	2.39 (0.477)	2.50 (0.428)	2.01 (0.344)
CV%	22.6	19.9	17.1	17.1
Median (min, max)	2.81 (1.98, 4.30)	2.37 (1.79, 3.60)	2.38 (1.82, 3.31)	2.01 (1.15, 2.66)

SD, standard deviation; CV%, percentage of covariance; a.m.: the morning time; p.m.: the afternoon to evening time.

**Table 3:** Mean (SD) of BH_4_ and sepiapterin PK parameters in non-Japanese and Japanese participants.

Parameter	Sepiapterin (20 mg/kg)		Sepiapterin (40 mg/kg)			Sepiapterin (60 mg/kg)	
	Non-Japanese Fed	Japanese Fed	Non-Japanese Fed	Japanese Fed	Japanese Fasted	Non-Japanese Fed	Japanese Fed
	(*n* = 6)	(*n* = 6)	(*n* = 6)	(*n* = 4)^d^	(*n* = 6)	(*n* = 6)	(*n* = 6)
BH_4_							
AUC_0–last_, h·ng/mL	2670 (150)	2970 (607)	2830 (662)	3490 (772)	2200 (311)	2720 (491)	3290 (234)
AUC_0–inf_, h·ng/mL	2710 (140)	3100 (660) ^b^	2790 (679) ^b^	3390 (885) ^c^	2370 (500) ^c^	2580 (487) ^c^	3260 (205) ^d^
C_max_, ng/mL	420 (37)	485 (109)	438 (99)	567 (148)	340 (46)	403 (81)	514 (60)
T_max_ ^a^, h	4.0 (4.0, 5.0)	5.0 (4.0, 5.0)	4.5 (3.0, 5.0)	5.0 (4.1, 5.0)	5.0 (4.0, 5.0)	4.0 (3.0, 5.0)	4.0 (3.0, 5.0)
T_1/2_, h	6.07 (0.941)	6.35 (0.781) ^b^	7.78 (2.08) ^b^	6.88 (1.33) ^c^	9.83 (2.14) ^c^	7.57 (1.59) ^c^	6.78 (1.44) ^d^
Sepiapterin							
AUC_0–last_, h·ng/mL	8.1 (3.5)	11.8 (4.3)	13.3 (9)	15.9 (4.8)	10.3 (4.2)	13.1 (6.9)	25.3 (12.8)
C_max_, ng/mL	2.1 (0.8)	2.4 (0.8)	2.7 (1.2)	3.4 (0.4)	2.7 (0.9)	2.4 (1)	4.9 (1.5)
T_max_^a^, h	3 (1.0, 4.0)	4.5 (3.0, 5.0)	3.5 (2.0, 12.0)	3 (2.0, 5.0)	1 (0.5, 3.0)	2 (1.0, 4.0)	4 (2.0, 5.0)

AUC_0–inf_, area under the concentration–time curve from time zero extrapolated to infinity; AUC_0–last_, area under the plasma concentration–time curve from the start of dosing to the last quantifiable concentration; BH_4_, tetrahydrobiopterin; C_max_, maximum observed plasma concentration; PK, pharmacokinetics; SD, standard deviation; T_1/2_, apparent terminal elimination half-life; T_max_, time to maximum observed plasma concentration. ^a^ T_max_ is presented as median (minimum, maximum). ^b^ *n* = 5. ^c^ *n* = 3. ^d^ *n* = 4. Two participants were excluded from the analysis due to deviation from the low-fat diet on the day of dosing.

**Table 4:** Geometric mean ratios of BH_4_ exposures in Japanese and non-Japanese participants following a single oral dose of sepiapterin.

Parameter	Sepiapterin Dose	BH_4_ GLSM		BH_4_ GMR, % (90% CI)
		Non-Japanese (Reference)	Japanese (Test)	
AUC_0–last_ (h·ng/mL)	20 mg/kg	2663	2920	110 (92–130)
	40 mg/kg	2762	3422	124 (102–150)
	60 mg/kg	2685	3284	122 (103–145)
AUC_0–inf_ (h·ng/mL)	20 mg/kg	2709	3002	111 (93–132)
	40 mg/kg	2724	3315	122 (98–152)
	60 mg/kg	2552	3256	128 (101–161)
C_max_ (ng/mL)	20 mg/kg	419	476	114 (94–137)
	40 mg/kg	429	552	129 (104–159)
	60 mg/kg	396	511	129 (107–156)

AUC_0–inf_, area under the concentration–time curve from time zero extrapolated to infinity; AUC_0–last_, area under the plasma concentration–time curve from the start of dosing to the last quantifiable concentration; BH_4_, tetrahydrobiopterin; CI, confidence interval; C_max_, maximum observed plasma concentration; GLSM, geometric least square mean; GMR, geometric mean ratio.

**Table 5:** Effects of food on BH_4_ exposures in Japanese participants following a single dose of 40 mg/kg sepiapterin.

Parameter	BH_4_ GLSM		BH_4_ GMR % (90% CI)
	Fasted (Reference)	Fed ^a^ (Test)	
	(*n* = 4)	(*n* = 4)	
AUC_0–last_ (h·ng/mL)	1962	3290	168 (122–230)
C_max_ (ng/mL)	320	532	167 (109–254)

AUC_0–last_, area under the plasma concentration–time curve from the start of dosing to the last quantifiable concentration; BH_4_, tetrahydrobiopterin; CI, confidence interval; C_max_, maximum observed plasma concentration; GLSM, geometric least square mean; GMR, geometric mean ratio. ^a^ Fed = low-fat low-calorie diet.

**Table 6:** Incidence of treatment-emergent adverse events.

	Incidence, Number of Participants (%)/Number of TEAEs							
	Cohorts 1 and 4 Fed Non-Japanese	Cohorts 1 and 4 Fed Japanese	Cohorts 2 and 5 Fed Non-Japanese	Cohorts 2 and 5 Fasted Japanese	Cohorts 2 and 5 Fed Japanese	Cohort 3 Fed Non-Japanese	Cohort 3 Fed Japanese	Overall
	20 mg/kg		40 mg/kg			60 mg/kg		
*n*	12	12	12	12	12	6	6	60
Any TEAE	2 (16.7)/3	4 (33.3)/4	3 (25.0)/8	5 (41.7)/5	4 (33.3)/9	1 (16.7)/3	1 (16.7)/1	18 (30.0)/33
Mild	2 (16.7)/2	4 (33.3)/4	3 (25.0)/6	5 (41.7)/5	4 (33.3)/8	1 (16.7)/2	1 (16.7)/1	18 (30.0)/28
Moderate	1 (8.3)/1	0	2 (16.7)/2	0	1 (8.3)/1	1 (16.7)/1	0	5 (8.3)/5
Severe	0	0	0	0	0	0	0	0
Life-threatening	0	0	0	0	0	0	0	0
TEAE related to drug	1 (8.3)/1	3 (25.0)/3	3 (25.0)/7	4 (33.3)/4	2 (16.7)/4	1 (16.7)/1	1 (16.7)/1	14 (23.3)/21
TEAE leading to discontinuation	0	0	0	0	0	0	0	0

TEAE, treatment-emergent adverse event.

## Data Availability

The datasets generated and/or analyzed during this current study are available from the corresponding author upon reasonable request.
